# Different methods of killing bacteria diets differentially influence *Caenorhabditis elegans* physiology

**DOI:** 10.17912/micropub.biology.000902

**Published:** 2023-09-07

**Authors:** Nicole L. Stuhr, Sean P. Curran

**Affiliations:** 1 Molecular and Computational Biology, University of Southern California, Los Angeles, California, United States; 2 Leonard Davis School of Gerontology, University of Southern California, Los Angeles, California, United States

## Abstract

Across species, diet plays a critical role in most, if not all life history traits. *Caenorhabditis elegans* is an important and facile organism for research across modalities, but the use of live bacteria as sources of nutrition can exert pleiotropic outcomes that stem from the action of host-pathogen defenses. Recently, a powerful new approach to readily generate dead and metabolically inactive *Escherichia coli* was developed that enabled reproducible measures of health across the lifespan. Here we further characterize additional comparisons of developmental and physiological parameters of animals fed either bacteria killed by treatment with ultraviolet (UV) light and bactericidal antibiotics or low-dose paraformaldehyde (PFA). Unlike bacteria killed by UV/Antibiotic treatment, PFA-killed diets resulted in a 25% reduction in body size just prior to adulthood and an overall reduction in stored intracellular lipids. Moreover, a small but reproducible number of animals fed PFA-killed bacteria display age-dependent depletion of somatic lipids, which does not normally occur on live bacteria or bacteria killed by UV/antibiotics. Lastly, animals fed PFA-treated, but not UV-antibiotic treated bacteria display a 10% increase in crawling speed. Taken together, these new data more thoroughly define the physiological impact two methodologies to prepare *C. elegans* diets that should be considered during experimental design.

**Figure 1. Differential effects of PFA-killed and UV/Antibiotic-killed E. coli on C. elegans physiology:**
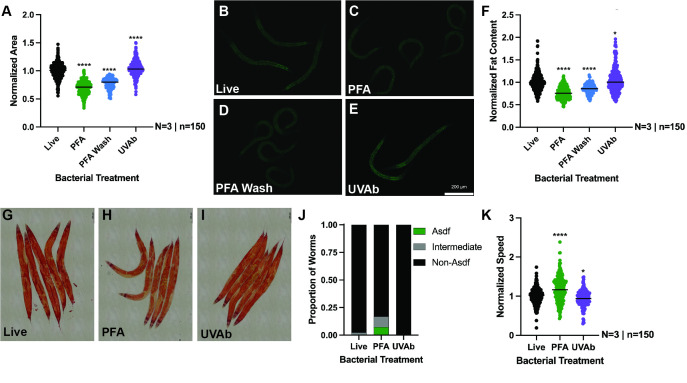
The method of killing the *C. elegans* OP50 diet prior to feeding can differentially affect animal growth (A), stored intracellular lipids as visualized by fixed Nile red staining (B-E) and normalized to body area (F), distribution of stored lipid pools as visualized by fixed oil-red-o staining (G-J), and animal movement speed in early life (K).

## Description

The metabolic activity of live bacteria as a food source can significantly impact physiological outcomes in animals fed these diets, particularly in pharmacological studies and when the goal is to understand the complexities of metabolism and nutrition (Garigan, Hsu et al. 2002, Yen and Curran 2016, Qi, Kniazeva et al. 2017) . Although multiple methods have previously been reported several techniques have limitations that confound the interpretation of the data; for example, heat-killed bacteria can be difficult for *C. elegans* to ingest and results in calorie restricted state that changes developmental timing (Qi, Kniazeva et al. 2017) , UV irradiated bacteria is low throughput and results in incomplete and inconsistent killing (Gems and Riddle 2000) , yet still can influence the lifespan of *C. elegans* (Garigan, Hsu et al. 2002, Cabreiro, Au et al. 2013, Sanchez-Blanco, Rodriguez-Matellan et al. 2016) , and antibiotic treatment can differ based on the bactericidal or bacteriolytic nature of the drug and also results in altered lifespan (Garigan, Hsu et al. 2002, Cabreiro, Au et al. 2013) . The effects on lifespan are intriguing but add an additional variable of host-pathogen responses that can make uncoupling the effects of genetics, pharmacological treatment, or nutritional changes more difficult.

A recent study optimized an effective and reproducible method for killing bacteria using paraformaldehyde (PFA) at low doses (Beydoun, Choi et al. 2021) . This study highlights not only the consistency of killing bacteria, but also demonstrates that PFA-killed bacteria do not replicate but are also metabolically inactive. Importantly, despite PFA-killed *E. coli* slightly delaying larval development to adulthood, other life history traits like reproduction and lifespan, are not changed (Beydoun, Choi et al. 2021) , suggesting worms are healthy when fed this preparation of food. One parameter missing from this otherwise thorough investigation of the impact that PFA-killed *E. coli* has on *C. elegans* health is an assessment of lipid homeostasis and additional healthspan measures such as movement speed; here we provide additional data to fill this knowledge gap.

We investigated these parameters in *C. elegans* fed an *E. coli* diet killed by UV/antibiotic (UVAb) exposure as compared to a low dose (0.25%) PFA treatment. We confirmed the slight developmental delay reported in animals fed PFA-killed OP50 , but subsequently compared animals at the L4 development stage for overall body size. We found that when compared to animals fed live OP50 and UVAb killed OP50, animals raised on PFA-killed *E. coli* OP50 were significantly smaller at the L4 larval stage ( **Figure 1A** ). We next performed fixed Nile red staining to quantify the abundance of intracellular lipid pools, which revealed animals fed PFA-killed OP50 stored less lipids relative to animals fed live bacteria ( **Figure 1B -F** ). Notably, the magnitude of this difference is more significant when compared to animals fed UV/antibiotic-killed bacteria, which display a modest increase in intracellular lipid pools. We confirmed that the growth and lipid storage phenotypes were not due to the presence of residual PFA in the preparation of the bacterial, because the decrease in body size and lipid content persists when the PFA-killed bacteria are prepared with additional washes with M9 buffer (PFA Wash). Bacterial diets can also influence where lipids are distributed between somatic and germ tissues (Lynn, Dalton et al. 2015, Nhan, Turner et al. 2019) , and as such, we also performed Oil-Red-O staining to qualitatively assess the location of lipid stores in animals with age. Worms fed PFA-killed *E. coli* display a small but significant increase in the incidence of somatic depletion of fat, while maintaining germline lipid pools at day 4 of adulthood ( **Figure 1G -J** ). This result is intriguing because depletion of somatic lipid stores can also occur when animals are exposed to pathogenic bacteria (Nhan, Turner et al. 2019) . Taken together, these data reveal that suggests that despite PFA-killed bacteria being metabolically inactive, when provided as the sole source of nutrition to *C. elegans* it maintains the capacity to induce metabolic change.

Finally, we tested how the different dead bacterial diets can influence overall health by measuring movement speed as a surrogate for muscle function (Roussel, Sprenger et al. 2014) . We measured the average speed of wildtype worms at the L4 stage on plates without *E. coli* after being raised on either live OP50 or killed OP50 from the L1 to L4 larval stages ( **Figure 1K** ). We found that raising worms on PFA-treated OP50 led to a significant increase in average crawling speed. This increase in crawling speed was not observed in animals raised on the UV/Antibiotic-killed OP50.

Taken together, our results show that presenting live or dead bacterial diets to *C. elegans* can influence metabolism and healthspan; two phenotypes that require further investigation in the context of diet as most studies have exclusively used live bacteria (Garigan, Hsu et al. 2002, Cabreiro, Au et al. 2013, Scott, Quintaneiro et al. 2017, Beydoun, Choi et al. 2021) *.* These results fill knowledge gaps in the field and emphasize the importance of bacterial respiration on *C. elegans* physiology which should be taken into consideration for study design.

## Methods

***C. elegans* strains and maintenance**

*C. elegans* were raised on 6 cm nematode growth media (NGM) plates supplemented with streptomycin and seeded with live OP50 . For experiments, nematode growth media plates without streptomycin were seeded with live OP50 , UV/Antibiotic-killed OP50 or PFA-killed bacteria. The N2 Bristol (wildtype) worm strain was grown at 20°C and unstarved for at least three generations before being used. Some strains were provided by the CGC, which is funded by NIH Office of Research Infrastructure Programs (P40 OD010440).

**Killed-OP50 Treatments**

After culturing, 400 mL of live bacteria was aliquoted into 1000mL Erlenmeyer flasks. The first flask contained live OP50 . The second and third flask had 10% PFA was added to the second flask to get the desired final concentration of PFA (0.25% PFA). PFA-treated bacteria was shaken at 37 °C in a shaking incubator at 200 rpm for 2 hours to allow for mixing of PFA and sufficient exposure. The fourth flask had 4% kanamycin and 4% ampicillin was added to the culture and incubated in a shaking incubator at 200 rpm for 12 hours. All conditions were then spun down and resuspended in M9 at 1x concentration for live OP50 and 5x concentration for PFA-treated and UV/Antibiotic-treated OP50 . The second flask (PFA-treated bacteria) was washed one additional time in M9 before resuspension. The “PFA Wash” condition (third flask) had two subsequent wash steps in M9 before resuspension at 5x concentration. UV/Antibiotic-killed OP50 (fourth flask) was then exposed to 25000 mJ of UV before all bacteria were seeded on NGM plates and left to dry for 48 hours before the experiments. Previous studies have used 0.5% PFA exposure for PFA-killed bacteria resuspended in LB ^6^ . We adapted this protocol and used 0.25% PFA exposure with resuspension of bacteria in M9.

**Nile Red Staining**

Nile Red fat staining was conducted as previously described [12, 13]. In brief, worms were egg prepped and allowed to hatch overnight for a synchronous L1 population. The next day, worms were dropped onto plates seeded with bacteria and raised to 48 h (L4 stage). Worms were washed off plates with PBS+triton, rocked for 3 min in 40% isopropyl alcohol before being pelleted and treated with Nile Red in 40% isopropyl alcohol for 2 h. Worms were pelleted after 2 h and washed in PBS+triton for 30 min before being images at 10X magnification with ZEN Software and Zen Axio Imager with the DIC and GFP filter. Fluorescence is measured via corrected total cell fluorescence (CTCF) via ImageJ and Microsoft Excel as previously described [14]. CTCF = Worm Integrated Density-(Area of selected cell X Mean fluorescence of background readings) and normalized to the control.

**Oil Red O Staining**

Oil Red O fat staining was conducted as outlined [15]. In brief, worms were egg prepped and allowed to hatch overnight for a synchronous L1 population. The next day, worms were dropped onto plates seeded with bacteria and raised to 120 h (Day 3 Adult stage). Worms were washed off plates with PBS+triton, then rocked for 3 min in 40% isopropyl alcohol before being pelleted and treated with ORO in diH2O for 2 h. Worms were pelleted after 2 h and washed in PBS+triton for 30 min before being imaged at 20x magnification with LAS X software and Leica Thunder Imager flexacam C3 color camera.

**Lipid distribution**

ORO-stained worms were placed on glass slides and a coverslip was placed over the sample. Worms were scored, as previously described [15]; the age-dependent somatic depletion of fat (Asdf) phenotype is characterized by the loss of detection of ORO-stained lipids in the soma but presence of lipids in the germline. Worms were scored and images were taken with LAS X software and Leica Thunder Imager flexacam C3 color camera. Fat levels of worms were placed into two categories: non-Asdf and Asdf. Non-Asdf worms display no loss of fat and are stained dark red throughout most of the body (somatic and germ cells). Asdf worms had most, if not all, observable somatic fat deposits depleted (germ cells only) or significant fat loss from the somatic tissues with portions of the intestine being clear (somatic < germ).

**Movement Measurements – Crawling**

Worms were egg prepped and eggs were allowed to hatch overnight for a synchronous L1 population. The next day, worms were dropped onto plates seeded with bacteria. Worms were then allowed to grow until each time point (48 h post-drop for L4s). Once worms were the required stage of development, 30-50 worms were washed off of a plate in 50 uL of M9 with a M9+triton coated P1000 tip and dropped onto an unseeded NGM plate as previously described[16]. The M9 was allowed to dissipate, and worms roamed on the unseeded plate for 1 hour before imaging crawling. Crawling was imaged with the MBF Bioscience WormLab microscope and analysis was performed with WormLab version 2022. Worm crawling on the plate was imaged for 1 minute for each condition at 7.5 ms. Worm crawling was analyzed with the software and only worms that moved for at least 90% of the time were included in the analysis.
